# Supplementary material for: Comparison of the disinfecting effect of sodium hypochlorite aqueous solution and surfactant on hospital kitchen hygiene using adenosine triphosphate swab testing
Source: PLoS One. 2021 Apr 13;16(4):e0249796. doi: 10.1371/journal.pone.0249796 (PMC8043410; doi:10.1371/journal.pone.0249796)
Supplement: Attachment — Submitted filename: PONE-D-20-31068_reviewer KITCHEN.pdf [file pone.0249796.s002.pdf]

PLOS ONE

Comparison of the disinfecting effect of sodium hypochlorite aqueous solution and surfactant on hospital kitchen hygiene using adenosine triphosphate swab testing

--Manuscript Draft--

Manuscript Number:	PONE-D-20-31068
Article Type:	Research Article
Full Title:	Comparison of the disinfecting effect of sodium hypochlorite aqueous solution and surfactant on hospital kitchen hygiene using adenosine triphosphate swab testing
Short Title:	Comparing disinfection between HYP and surfactant using ATP swab testing
Corresponding Author:	Takashi Aoyama, Ph.D.<br>Shizuoka Cancer Center: Shizuoka Kenritsu Shizuoka Gan Center<br>Shizuoka, JAPAN
Keywords:	
Abstract:	The Japanese Ministry of Health requires large-scale cooking facilities to use sodium hypochlorite aqueous solution (HYP) on food preparation tools, equipment, and facilities to prevent secondary contamination. This study aimed to compare the disinfecting effect of HYP and surfactant using adenosine triphosphate (ATP) swab testing on large-scale equipment and facilities in hospital kitchens that could not be disassembled and disinfected. From May 2018 to July 2018, ATP swab tests were performed on the following six locations in the Shizuoka Cancer Centre Dietary Department Kitchen: cooking counter, mobile cooking counter, refrigerator handle, conveyor belt, tap handle, and sink. Six relative light unit (RLU) measurements were taken from each location. The log 10 values of the RLU measurements were evaluated by dividing the samples into two groups: the Control group (surfactant followed by HYP swabbing) and the HYP group (HYP swabbing only). The results showed that the RLUs (log 10 values) in both the groups improved after disinfection (p <0.05), except for the RLUs (log 10 values) of the mobile cooking counter, tap handle, and sink in the Control group after the HYP swab. The changes in the RLU (log 10 value) did not differ between the two groups for all locations of the kitchen. Hence, HYP swabbing of large-scale equipment and facilities provides the same level of disinfecting effect as surfactants and can be as beneficial.
Order of Authors:	Takashi Aoyama, Ph.D.
	Tomoko Kudo
Additional Information:	
**Question**	**Response**
**Financial Disclosure**<br><br>Enter a financial disclosure statement that describes the sources of funding for the work included in this submission. Review the submission guidelines for detailed requirements. View published research articles from *PLOS ONE* for specific examples.<br><br>This statement is required for submission and **will appear in the published article** if the submission is accepted. Please make sure it is accurate.	The author(s) received no specific funding for this work.

Powered by Editorial Manager® and ProduXion Manager® from Aries Systems Corporation

**Unfunded studies**<br>Enter: *The author(s) received no specific funding for this work.*<br><br>**Funded studies**<br>Enter a statement with the following details:<br>• Initials of the authors who received each award<br>• Grant numbers awarded to each author<br>• The full name of each funder<br>• URL of each funder website<br>• Did the sponsors or funders play any role in the study design, data collection and analysis, decision to publish, or preparation of the manuscript?<br>• **NO** - Include this sentence at the end of your statement: *The funders had no role in study design, data collection and analysis, decision to publish, or preparation of the manuscript.*<br>• **YES** - Specify the role(s) played.<br><br>* typeset	
**Competing Interests**<br><br>Use the instructions below to enter a competing interest statement for this submission. On behalf of all authors, disclose any competing interests that could be perceived to bias this work—acknowledging all financial support and any other relevant financial or non-financial competing interests.<br><br>This statement **will appear in the published article** if the submission is accepted. Please make sure it is accurate. View published research articles from *PLOS ONE* for specific examples.	NO authors have competing interests

**NO authors have competing interests**<br><br>Enter: *The authors have declared that no competing interests exist.*<br><br>**Authors with competing interests**<br><br>Enter competing interest details beginning with this statement:<br><br>*I have read the journal's policy and the authors of this manuscript have the following competing interests: [insert competing interests here]*<br><br>* typeset	
**Ethics Statement**<br><br>Enter an ethics statement for this submission. This statement is required if the study involved:<br><br>• Human participants<br>• Human specimens or tissue<br>• Vertebrate animals or cephalopods<br>• Vertebrate embryos or tissues<br>• Field research<br><br>Write "N/A" if the submission does not require an ethics statement.<br><br>General guidance is provided below. Consult the submission guidelines for detailed instructions. **Make sure that all information entered here is included in the Methods section of the manuscript.**	Field research

Powered by Editorial Manager® and ProduXion Manager® from Aries Systems Corporation

**Format for specific study types**<br><br>**Human Subject Research (involving human participants and/or tissue)**<br>• Give the name of the institutional review board or ethics committee that approved the study<br>• Include the approval number and/or a statement indicating approval of this research<br>• Indicate the form of consent obtained (written/oral) or the reason that consent was not obtained (e.g. the data were analyzed anonymously)<br><br>**Animal Research (involving vertebrate animals, embryos or tissues)**<br>• Provide the name of the Institutional Animal Care and Use Committee (IACUC) or other relevant ethics board that reviewed the study protocol, and indicate whether they approved this research or granted a formal waiver of ethical approval<br>• Include an approval number if one was obtained<br>• If the study involved *non-human primates*, add *additional details* about animal welfare and steps taken to ameliorate suffering<br>• If anesthesia, euthanasia, or any kind of animal sacrifice is part of the study, include briefly which substances and/or methods were applied<br><br>**Field Research**<br><br>Include the following details if this study involves the collection of plant, animal, or other materials from a natural setting:<br>• Field permit number<br>• Name of the institution or relevant body that granted permission	
**Data Availability**<br><br>Authors are required to make all data underlying the findings described fully available, without restriction, and from the time of publication. PLOS allows rare exceptions to address legal and ethical concerns. See the PLOS Data Policy and FAQ for detailed information.	Yes - all data are fully available without restriction

Powered by Editorial Manager® and ProduXion Manager® from Aries Systems Corporation

A Data Availability Statement describing where the data can be found is required at submission. Your answers to this question constitute the Data Availability Statement and **will be published in the article**, if accepted.<br><br>**Important:** Stating 'data available on request from the author' is not sufficient. If your data are only available upon request, select 'No' for the first question and explain your exceptional situation in the text box.<br><br>Do the authors confirm that all data underlying the findings described in their manuscript are fully available without restriction?	
**Describe where the data may be found in full sentences. If you are copying our sample text, replace any instances of XXX with the appropriate details.**<br><br>• If the data are **held or will be held in a public repository**, include URLs, accession numbers or DOIs. If this information will only be available after acceptance, indicate this by ticking the box below. For example: *All XXX files are available from the XXX database (accession number(s) XXX, XXX.*).<br>• If the data are all contained **within the manuscript and/or Supporting Information files**, enter the following: *All relevant data are within the manuscript and its Supporting Information files.*<br>• If neither of these applies but you are able to provide **details of access elsewhere**, with or without limitations, please do so. For example:<br><br>*Data cannot be shared publicly because of [XXX]. Data are available from the XXX Institutional Data Access / Ethics Committee (contact via XXX) for researchers who meet the criteria for access to confidential data.*<br><br>*The data underlying the results presented in the study are available from (include the name of the third party*	None

and contact information or URL).<br>• This text is appropriate if the data are owned by a third party and authors do not have permission to share the data.<br><br>* typeset	
Additional data availability information:	Tick here if your circumstances are not covered by the questions above and you need the journal’s help to make your data available.

Powered by Editorial Manager® and ProduXion Manager® from Aries Systems Corporation

Manuscript Click here to access/download;Manuscript;ATP One.docx

Comparison of the disinfecting effect of sodium hypochlorite aqueous solution and surfactant on hospital kitchen hygiene using adenosine triphosphate swab testing

Short title: Comparing disinfection between HYP and surfactant using ATP swab testing

Takashi Aoyama Tomoko Kudo

Dietary Department, Shizuoka Cancer Centre, Shizuoka, Japan

Division of Infection Control Nurse, Shizuoka Cancer Center, Japan

Corresponding author:

E-mail: t.aoyama@scchr.jp (TA)

Abstract

The Japanese Ministry of Health requires large-scale cooking facilities to use sodium hypochlorite aqueous solution (HYP) on food preparation tools, equipment, and facilities to prevent secondary contamination. This study aimed to compare the disinfecting effect of HYP and surfactant using adenosine triphosphate (ATP) swab testing on large-scale equipment and facilities in hospital kitchens that could not be disassembled and disinfected. From May 2018 to July 2018, ATP swab tests were performed on the following six locations in the Shizuoka Cancer Centre Dietary Department Kitchen: cooking counter, mobile cooking counter, refrigerator handle, conveyor belt, tap handle, and sink. Six relative light unit (RLU) measurements were taken from each location. The $\log_{10}$ values of the RLU measurements were evaluated by dividing the samples into two groups: the Control group (surfactant followed by HYP swabbing) and the HYP group (HYP swabbing only). The results showed that the RLUs ($\log_{10}$ values) in both the groups improved after disinfection ($p<0.05$), except for the RLUs ($\log_{10}$ values) of the mobile cooking counter, tap handle, and sink in the Control group after the HYP swab. The changes in the RLU ($\log_{10}$ value) did not differ between the two groups for all locations of the kitchen. Hence, HYP swabbing of large-scale equipment and facilities provides the same level of disinfecting effect as surfactants and can be as beneficial.

Introduction

The Japanese Ministry of Health, Labour and Welfare's "Hygienic Management

Manual for Large-Scale Cooking Facilities” applies to cooking facilities that provide 300 or more meals at a time or 750 or more meals in a day [1]. These measures comply with the Hazard Analysis and Critical Control Points (HACCP) principles and state that food preparation tools (e.g., cutting boards) should be soaked in sodium hypochlorite aqueous solution (200 ppm) (HYP), or when soaking is difficult, the same agent should be used to wipe the equipment and facilities to prevent secondary contamination by microbes such as norovirus [2, 3]. Large immovable equipment is easier to disinfect with HYP. Since the use of surfactants requires water, it also requires additional labor to maintain a dry floor surface. Therefore, the Shizuoka Cancer Centre Dietary Department Kitchen (SCC Kitchen) applies the same disinfecting agent to large equipment and facilities that cannot be disassembled and disinfected. While the manual does not require the testing of the hygiene status of large equipment and facilities, the SCC Dietary Department has introduced the use of relative light unit (RLU) values measured by adenosine triphosphate (ATP) swab testing [4], which has been shown to correlate with the colony-forming unit values of pre-disinfecting microbial cultures [5]. Previously, it was reported that ATP does not react with HYP [6] and that the hypochlorite ion (ClO^-) in HYP has a disinfecting effect [7]. In light of these reports, it was hypothesized that the disinfecting effect of HYP and surfactants on large equipment and facilities in hospital kitchens could be compared using ATP swab tests. However, there are several disadvantages associated with the use of HYP such as: (a) contamination with foreign bodies (organic substances) which quickly deactivates HYP, (b) HYP can decompose depending on the storage temperature and light exposure, (c) it cannot be prepared in advance, (d) it causes roughening of workers’ hands and has other handling issues such as the effort required in preparing diluted solutions, (e) unstable effects such as metal corrosion, and (f) adverse effects on

在這裡輸入文字

the respiratory tract [8]. In addition, no cleaning effect has been reported in an aqueous solution of sodium hypochlorite; however, it has been shown to have a disinfecting effect on the severe acute respiratory syndrome coronavirus 2 (SARS-CoV-2), which is responsible for the COVID-19 infection [9]. The purpose of this study was to compare the disinfecting effect of HYP and surfactant on large equipment and facilities using ATP
It is ..
swab testing and to explore the benefits of HYP. It was hypothesized that the disinfecting effect of HYP swabbing of large-scale cooking facilities may outweigh its operational disadvantages, as long as it is properly handled.

Materials and methods

Examination of the disinfecting effect of HYP

DELETE .. The ..

The ATP swab tests were conducted on random days between May 2018 and July
HOW MANY TESTS?
2018. The SCC Dietary Department had a floor area of 712.05 m^2 (including rest rooms), had six hospital staff and approximately 40 contracted staff, and served approximately 1,000 hospital meals. This study examined six locations (cooking counter, mobile cooking counter, refrigerator handle, conveyor belt, tap handle, and sink) with which food material and cooking staff come into contact most often, and thus, making the maintenance of strict hygiene of these locations necessary.

This study did not involve human subjects, and therefore ethics board approval and informed consent were not required. The experimental procedures using the RLU measurements from ATP swab testing are shown in Fig 1. The disinfecting effect was

examined by dividing the sample cases into two groups: the Control group that underwent the surfactant swab (70%; 10-fold dilution: pH 8.0, Advantec® Test Paper) followed by the HYP (200 ppm; pH: 9.4, Advantec® Test Paper) swab and the HYP group that underwent the HYP (200 ppm; pH: 9.4, Advantec® Test Paper) swab only.

在這裡輸入文字.

Fig 1. Evaluation of Cleaning/Disinfection by ATP Testing. Experimental procedures for cleaning and disinfecting large equipment and facilities using adenosine triphosphate (ATP) testing in the Shizuoka Cancer Centre Dietary Department Kitchen that could not be disassembled and disinfected.

ATP swab tests were performed at the end of the lunchtime meal distribution using conveyor belts (11:30 AM to 12:00 PM), with each of the six locations being tested a total of six times to obtain the RLU values: pre-disinfecting (A), post-surfactant swab (B), and post-HYP swab (C) for the Control group, and pre-disinfecting (ⓐ) and post-HYP swab (ⓒ) for the HYP group; this was done to evaluate the RLU values and $\log_{10}$ values (Control group: three specimens × six locations × six replicates; HYP group: two specimens × six locations × six replicates). The changes in the $\log_{10}$ of RLU values from the pre-disinfecting (A) to post-surfactant swab (B) for the Control group, and from the pre-disinfecting (ⓐ) to post-HYP swab (ⓒ) for the HYP group were compared. In addition, the changes in $\log_{10}$ of RLU values from the pre-disinfecting (A) to post-HYP swab (C) for the Control group and from the pre-disinfecting (ⓐ) to post-HYP swab (ⓒ) for the HYP group were compared. Lumitester®PD-20 (Kikkoman Biochemifa Company, Tokyo, Japan) was used for ATP swab testing. To perform the tests, the RLU value of

each test location was measured by swabbing a 10 cm^2 area of the test location (cooking counter: 10 cm^2 of all four counters; mobile cooking counter: 10 cm^2 of 15 counters; refrigerator handle: back and forth and front and back of eight handles; conveyor belt: 10 cm^2 of 20 m; tap handle: back and forth of eight handles; sinks: 10 cm^2 of the side walls

. This me

of four sinks) with a LuciPac® Pen soaked in tap water (residual chlorine concentration: 0.2 ppm, determined by a residual chlorine measuring reagent), in accordance with the method of measurement described in the manufacturer's product specifications. When the RLU value was zero, the measurement was re-taken, as it may not have been taken correctly initially.

Statistical analysis

The evaluation items are presented as median values (minimum–maximum), and normality was evaluated using the Shapiro–Wilk test. The RLU values were converted to a common logarithm (log_{10}) to obtain normality, and the changes in measurements before

control

and after disinfecting in the Control group and HYP group, as well as the differences in changes between the two groups, were evaluated using a paired Student's t-test. The statistical analysis software used was JMP® 12 (SAS Institute Inc., Cary, NC, USA). The results of statistical testing are shown using the 95% confidence interval, with a result below 0.05 ($p<0.05$) indicating statistical significance.

Results

The six test locations were tested six times each after the lunchtime meal

distribution for the Control group and HYP group by ATP swab testing (on May 23, 2018; June 6, 11, 18, and 24, 2018; and July 24, 2018). The median number of meals (range: minimum–maximum) was 367 meals (344–385; *p*=0.83), and the temperature and humidity in the kitchen were 22.5°C (20.8–24.1°C; *p*=0.86) and 66.0% (61.0–74.0%; *p*=0.70) respectively, which meant that experimental conditions related to the degree of contamination, including the number of meals provided, were in place. Table 1 shows the RLU and $\log_{10}$ values of the six test locations measured by ATP swab testing.

Table 1. Results of RLU values and $\log_{10}$ values determined by ATP testing (n=6).

			**Median**	**Minimum**	**Maximum**	p^a
**Cooking counter**	**RLU value**	A	15271	8277	58227	0.10
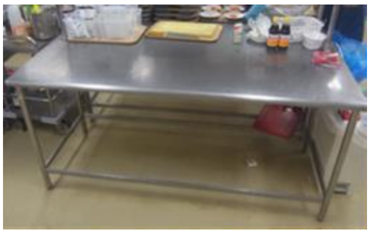		B	435	38	1191	0.29
		C	75	31	304	0.03
		ⓐ	37565	5539	115525	0.38
		ⓒ	283	17	916	0.10
	**$\log_{10}$ value**	A	4.1	3.9	4.8	0.15
		B	2.5	1.6	3.1	0.44
		C	1.9	1.5	2.5	0.60
		ⓐ	4.6	3.7	5.1	0.26
		ⓒ	2.2	1.2	3.0	0.23
**Mobile cooking counter**	**RLU value**	A	29250	2346	514254	<0.05
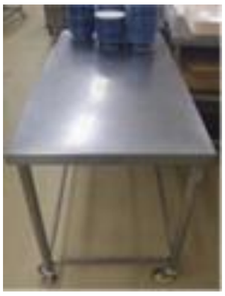		B	162	36	576	0.16
		C	88	27	214	0.02
		ⓐ	41513	2744	100474	0.75
		ⓒ	200	61	2471	0.01
	**$\log_{10}$ value**	A	4.4	3.4	5.7	0.86
		B	2.1	1.6	2.8	0.14
		C	1.9	1.4	2.3	0.22
		ⓐ	4.6	3.4	5.0	0.12
		ⓒ	2.3	1.8	3.4	0.55
**Refrigerator handle**	**RLU value**	A	4602	808	10358	0.55
		B	158	95	769	0.04
		C	42	14	378	0.01
		ⓐ	7921	2172	19092	0.75
		ⓒ	208	156	585	0.03
	**$\log_{10}$ value**	A	3.6	2.9	4.0	0.64
		B	2.2	2.0	2.9	0.18
		C	1.6	1.1	2.6	0.55

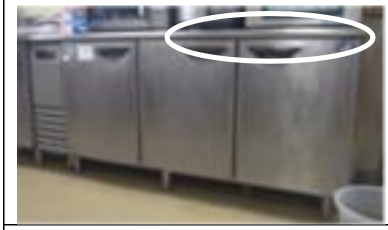		ⓐ	3.9	3.3	4.3	0.89
		ⓒ	2.3	2.2	2.8	0.08
**Conveyor belt**	**RLU value**	A	22938	8509	53190	0.49
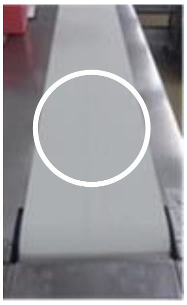		B	932	394	2791	0.21
		C	256	40	438	0.76
		ⓐ	21568	11079	46128	0.23
		ⓒ	391	189	5257	<0.05
	**log$_{10}$ value**	A	4.4	3.9	4.7	0.53
		B	3.0	2.6	3.4	0.85
		C	2.4	1.6	2.6	0.12
		ⓐ	4.3	4.0	4.7	0.58
		ⓒ	2.6	2.3	3.7	0.15
**Tap handle**	**RLU value**	A	10329	7868	29630	0.05
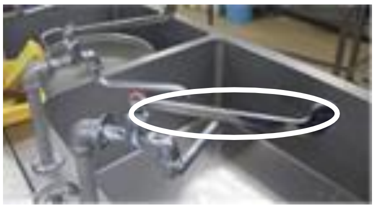		B	136	61	371	0.29
		C	97	38	561	0.01
		ⓐ	18104	5310	44148	0.43
		ⓒ	162	64	1574	<0.05
	**log$_{10}$ value**	A	4.0	3.9	4.5	0.23
		B	2.1	1.8	2.6	0.55
		C	2.0	1.6	2.7	0.16
		ⓐ	4.2	3.7	4.6	0.84
		ⓒ	2.2	1.8	3.2	0.34
**Interior of sink**	**RLU value**	A	2946	921	16134	0.08
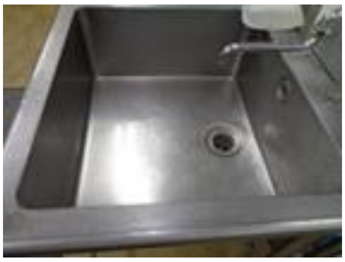		B	471	192	1075	0.52
		C	180	94	554	0.03
		ⓐ	11004	2278	61966	0.02
		ⓒ	639	263	975	0.57
	**log$_{10}$ value**	A	3.4	3.0	4.2	0.87
		B	2.7	2.3	3.0	0.79
		C	2.3	2.0	2.7	0.16
		ⓐ	3.9	3.4	4.8	0.07
		ⓒ	2.8	2.4	3.0	0.49

RLU, relative light unit; ATP, adenosine triphosphate

[a] *p* values were calculated by the Shapiro–Wilk test.

Table 2 and Figs 2-1, 2-2, and 2-3 show the comparison of RLU and log$_{10}$ values in the six test locations measured by ATP testing. In all the six test locations, there were significant improvements in the log$_{10}$ values of the RLUs from pre-disinfecting (A) to

post-surfactant swab (B) and post-HYP swab (C) for the Control group and from pre-disinfecting (ⓐ) to post-HYP swab (ⓒ) for the HYP group (p<0.05). There was no significant difference in the $\log_{10}$ values of the RLUs between post-surfactant swab (B) and post-HYP swab (C) for the Control group at the mobile cooking counter, tap handle, and sink (p=0.52, Fig 2-1 bottom; p=0.92, Fig 2-3 top; p=0.17, Fig 2-3 bottom); however, differences were observed at these time points for the Control group at the cooking counter, refrigerator handle, and conveyor belt (p<0.05). In all the six test locations, there were no significant differences in the changes of in the $\log_{10}$ values of the RLUs between post-HYP swab (ⓒ) of the HYP group and the post-surfactant swab (B) and post-HYP swab (C) of the control Control group (Figs 2-1, 2-2, and 2-3).

Fig 2-1. Inter-group comparison of the relative light unit (RLU) and $\log_{10}$ values in the cooking counter and mobile cooking counter test locations measured by adenosine triphosphate (ATP) swab testing. There were significant improvements in the $\log_{10}$ values of the RLUs from the pre-disinfecting (A) to post-surfactant swab (B) and post-HYP swab (C) for the control Control group, and from the pre-disinfecting (ⓐ) to post-HYP swab (ⓒ) for the HYP group (p<0.05). There was no significant difference in the $\log_{10}$ value of RLU values between the post-surfactant swab (B) and post-HYP swab (C) for the Control group at the mobile cooking counter. (p=0.52); however, differences were observed at these time points for the Control group at the cooking counter. In both these locations, there were no significant differences in the changes in the logarithmic RLU values between the post-HYP swab (ⓒ) of the HYP group and the post-surfactant swab (B) and post-HYP swab (C) of the Control group.

Fig 2-2. Inter-group comparison of the relative light unit (RLU) and $\log_{10}$ values in the refrigerator handle and conveyor belt test locations measured by adenosine triphosphate (ATP) swab testing. There were significant improvements in the $\log_{10}$ values of the RLUs from the pre-disinfecting (A) to post-surfactant swab (B) and the post-HYP swab (C) for the Control group, and from the pre-disinfecting (ⓐ) to post-HYP swab (ⓒ) for the HYP group (p<0.05). Significant differences in the $\log_{10}$ values of the RLUs between the post-surfactant swab (B) and post-HYP swab (C) for the Control group were observed the refrigerator handle and conveyor belt (p<0.05). In both these locations,
of the
there were no significant differences in the change in the $\log_{10}$ values of the RLUs between the post-HYP swab (ⓒ) of the HYP group and the post-surfactant swab (B) and post-HYP swab (C) of the Control group.

Fig 2-3. Inter-group comparison of the relative light unit (RLU) and $\log_{10}$ values in the tap handle and sink test locations measured by adenosine triphosphate (ATP) swab testing. There were significant improvements in the $\log_{10}$ values of the RLUs from the pre-disinfecting (A) to post-surfactant swab (B) and the post-HYP swab (C) for the Control group, and from the pre-disinfecting (ⓐ) to post-HYP swab (ⓒ) for the HYP group (p<0.05). There were no significant differences in the $\log_{10}$ values of the RLUs between the post-surfactant swab (B) and post-HYP swab (C) for the Control group at the tap handle and sink (p=0.92, Fig 2-3 top; p=0.17, Fig 2-3 bottom). In both these locations, there were no significant differences in the change in the $\log_{10}$ values of the RLUs between the post-HYP swab (ⓒ) of the HYP group and the post-surfactant swab (B) and

post-HYP swab (C) of the Control group.

Table 2. Results of the changes in the RLUs ($\log_{10}$ values) by ATP testing (n=6).

			Median: A	Minimum: A	Maximum: A
**Cooking counter**	**Change in $\log_{10}$ value**	A→B	-1.8	-3.1	-0.9
		A→.C	-2.5	-3.3	-1.4
		ⓐ→ⓒ	-2.4	-3.8	-0.8
**Mobile cooking counter**	**Change in $\log_{10}$ value**	A→B	-2.1	-3.3	-1.8
		A→.C	-2.6	-3.4	-1.54
		ⓐ→ⓒ	-1.9	-3.2	-1.4
**Refrigerator handle**	**Change in $\log_{10}$ value**	A→B	-1.5	-1.9	-0.02
		A→.C	-2.1	-2.7	-0.3
		ⓐ→ⓒ	-1.6	-2.0	-0.6
**Conveyor belt**	**Change in $\log_{10}$ value**	A→aB	-1.3	-2.0	-0.5
		A→.C	-2.0	-2.9	-1.5
		ⓐ→ⓒ	-1.7	-2.2	-0.3
**Tap handle**	**Change in $\log_{10}$ value**	A→aB	-1.9	-2.7	-1.4
		A→.C	-2.0	-2.3	-1.2
		ⓐ→ⓒ	-2.0	-2.5	-1.2
**Interior of sink**	**Change in $\log_{10}$ value**	A→aB	-0.9	-1.7	-0.1
		A→.C	-1.1	-2.2	-0.5
		ⓐ→ⓒ	-1.3	-2.1	-0.4

RLU, relative light unit; ATP, adenosine triphosphate

Discussion

While the Hygienic Management Manual for Large-Scale Cooking Facilities [1], which follows the HACCP, does not mandate the testing of environmental contamination, this study showed that the pre-disinfecting RLU values of large equipment and facilities in kitchens were as high as those that have been previously reported in other studies [5], thus making disinfection necessary (Table 1: A, ⓐ). Aside from the post-surfactant swab (B) and post-HYP swab (C) of the mobile cooking counter, tap handle, and sink for the Control group, all the $\log_{10}$ values of RLUs showed significant improvement, and it was shown that the disinfecting effect could be further improved by HYP swabbing for the cooking counter, refrigerator handle, and conveyor belt. There were no significant differences in the changes in the $\log_{10}$ values for both the groups. These results suggested that HYP swabbing for large equipment and facilities may have a disinfecting effect that is equivalent to that achieved by using surfactant. However, due to the instability and operational disadvantages associated with HYP (10%) in the SCC Kitchen, it is stored at 18°C and away from light before diluting to 200 ppm for use and requires handling with
TO WHOM???
disposable gloves. This study did not find any adverse events (respiratory problems, dermatological irritation, or metal corrosion) when this agent was used, and similar reports were not made by the National Consumer Affairs Centre [8, 10]. Hence, this study showed that the advantages of the disinfecting effect of HYP swabbing exceed its operational disadvantages, and that there may be benefits associated with using this agent. The aqueous solution of sodium hypochlorite has also been shown to be effective against enveloped viruses such as the SARS-CoV-2 [9]. These findings, which demonstrated the potential of HYP for general cleaning purposes, were considered to be significant.

This study has some limitations. It did not compare between surfactant and HYP in terms of resource costs and reduction in the amount of work required. In addition, this study did not explore the possibility of avoiding the wastage of human and water resources required for washing and the pollution caused by the waste. However, it should be noted that there have been very few studies that explored these possibilities.

In conclusion, this study used ATP swab testing to show that HYP swabbing
demonstrating
demonstrated a disinfecting effect similar to that of surfactant for large equipment and facilities in the hospital kitchen that cannot be disassembled and disinfected. Therefore, HYP, which has better disinfecting properties, may be a suitable disinfectant for such equipment.

Acknowledgements

the company
I would like to thank Editage (www.editage.com) for English language editing. I would also like to thank Naomi Katsumata, Makoto Suzukib, Keiko Mochizukib, and Tadashi Mochizukib who collected data for this study.

References

1. Ministry of Health, Labour and Welfare. Sanitary management of large scale cooking facilities manual. 2016 [Cited 2020 August 25]. Available from: https://www.mhlw.go.jp/file/06-Seisakujouhou-11130500-Shokuhinanzenbu/0000168026.pdf; 2016.
2. Ministry of Health, Labour and Welfare. Hazard Analysis and Critical Control Point. 2016 [Cited 2020 August 25]. Available from: https://www.mhlw.go.jp/stf/seisakunitsuite/bunya/kenkou_iryou/shokuhin/haccp/index_00002.html
3. Japan Society for Hematopoietic Stem Cell Transplantation. Guideline. 2017 [Cited 2020 August 25]. Available from: https://www.jshct.com/uploads/files/guideline/01_01_kansenkanri_ver04.pdf
4. Aoyama T, Tsukahara M, Kuroda H, Kurai H. Monitoring the hygienic status of hospital food preparation using adenosine triphosphate swab testing. Jpn J Environ Infect. 2017;32: 210-215. doi: 10.4058/jsei.32.210
5. Katsumata N, Aoyama T. Assessment of hospital kitchen hygiene using the adenosine triphosphate swab test and the direct contact plate method, and inter-method consistency. J Jpn Diet Assoc. 2018;61: 327-333. doi: 10.11379/jjda.61.327
6. Shinomiya S, Okubo T. On the effect of environmental disinfectants upon method of measuring adenosine tri-phosphate (ATP). J Healthc Assoc Infect 2014; 7: 7-23.
7. Fukuzaki S. Mechanisms of actions of sodium hypochlorite in Cleaning and

disinfection processes. Biocontrol Sci. 2006;11: 147-157. doi: 10.4265/bio.11.147

8. Shiraishi T, Nakagawa Y. Metal rusting by various disinfectants and influence of the rust on bactericidal effects. Environ Infect. 1999;14: 275-279. doi: 10.11550/jsei1986.14.275

9. National Consumer Affairs Centre of Japan (NCAC). 2020 [Cited 2020 August 25]. Available from: http://www.kokusen.go.jp/ncac_index_e.html

10. Ministry of Health, Labour and Welfare. About Coronavirus Disease 2019 (COVID-19). 2020 [Cited 2020 August 25]. Available from: https://www.mhlw.go.jp/stf/seisakunitsuite/bunya/syoudoku_00001.html

11.

Figure

Click here to access/download;Figure;Fig1.pptx

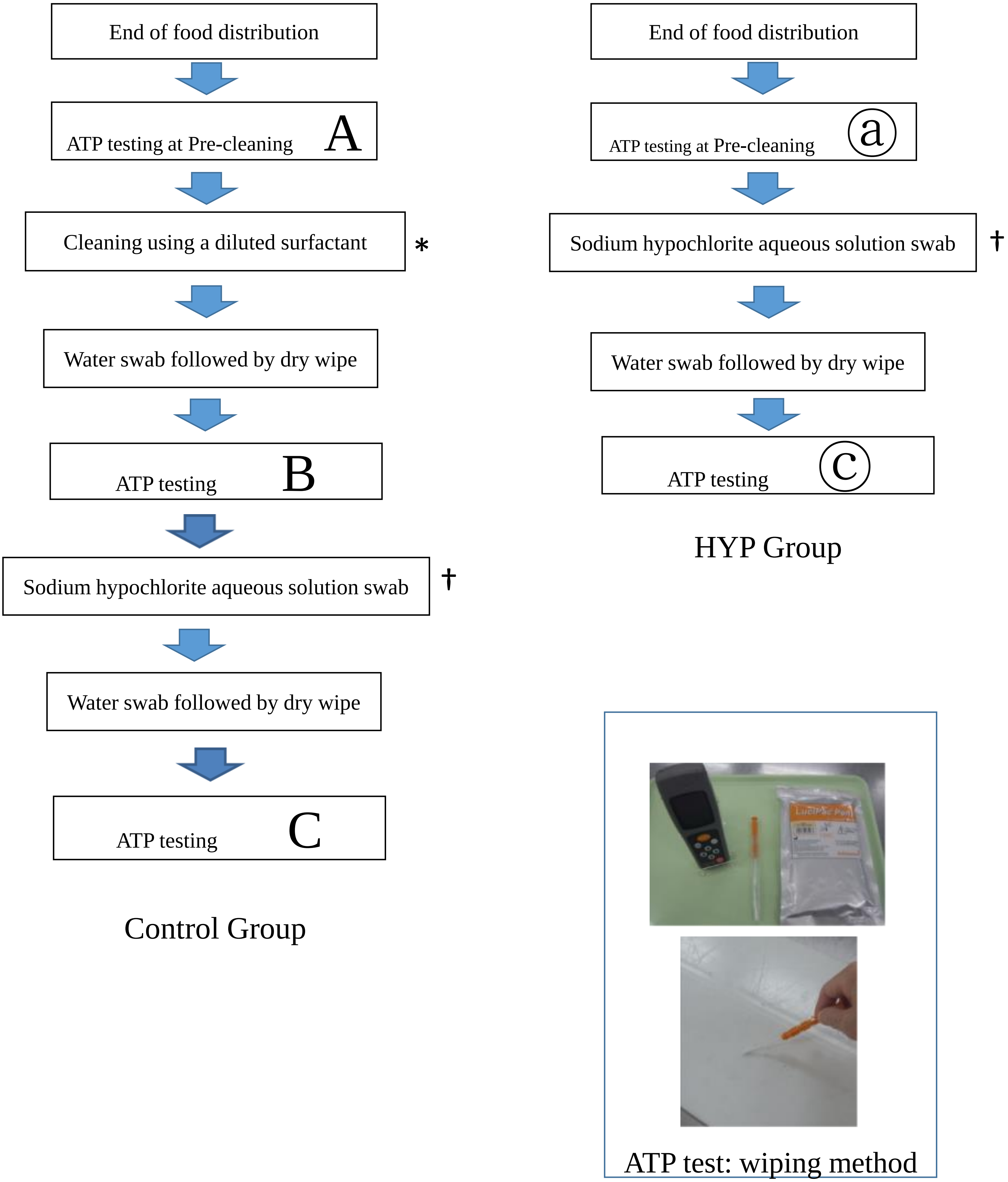

Fig. 1 Evaluation of Cleaning/Disinfection by ATP Testing

Click here to access/download;Figure;Fig2.pptx

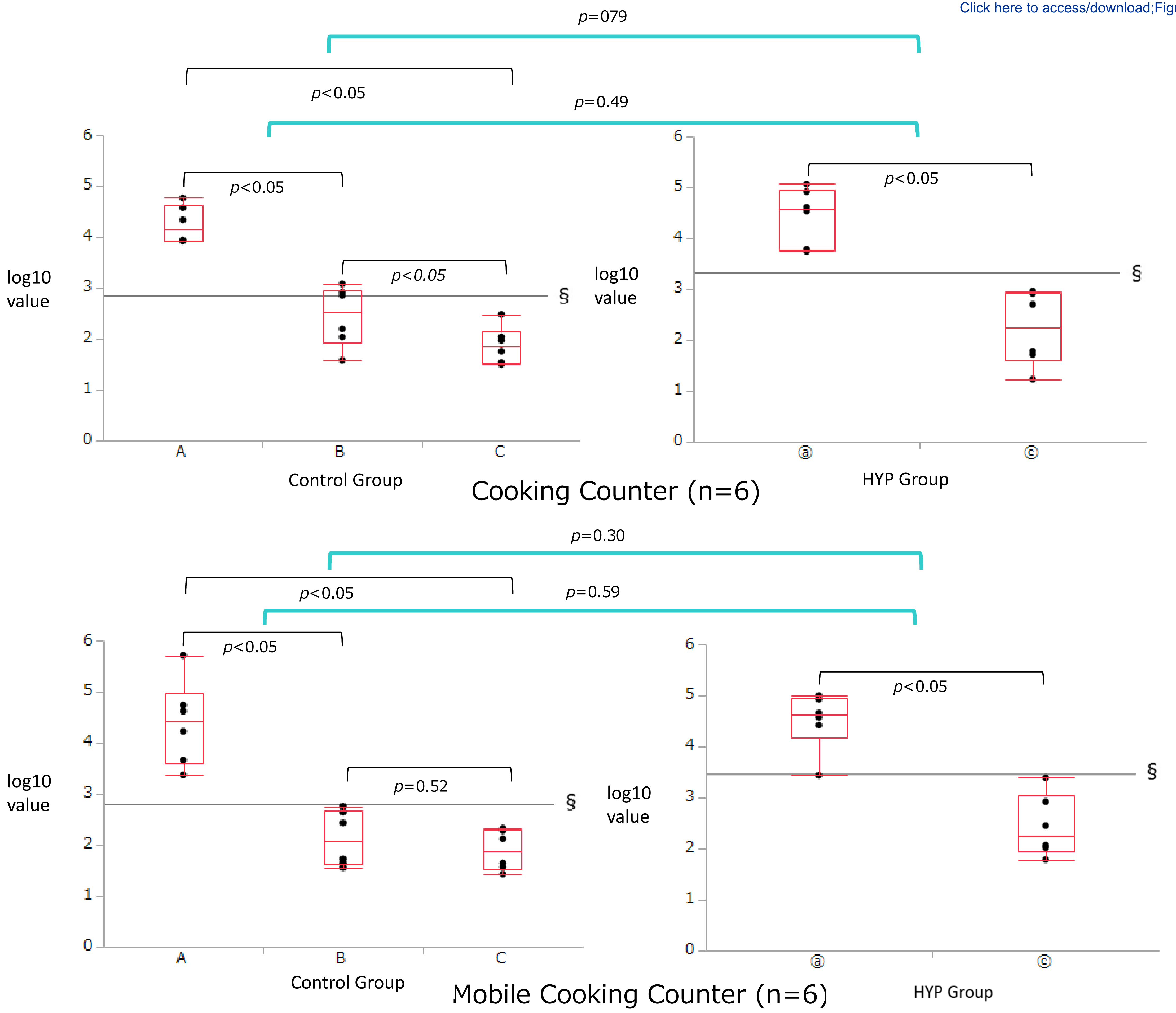

Fig. 2-1: Inter-group comparison of the log10 values in the 6 test locations measured

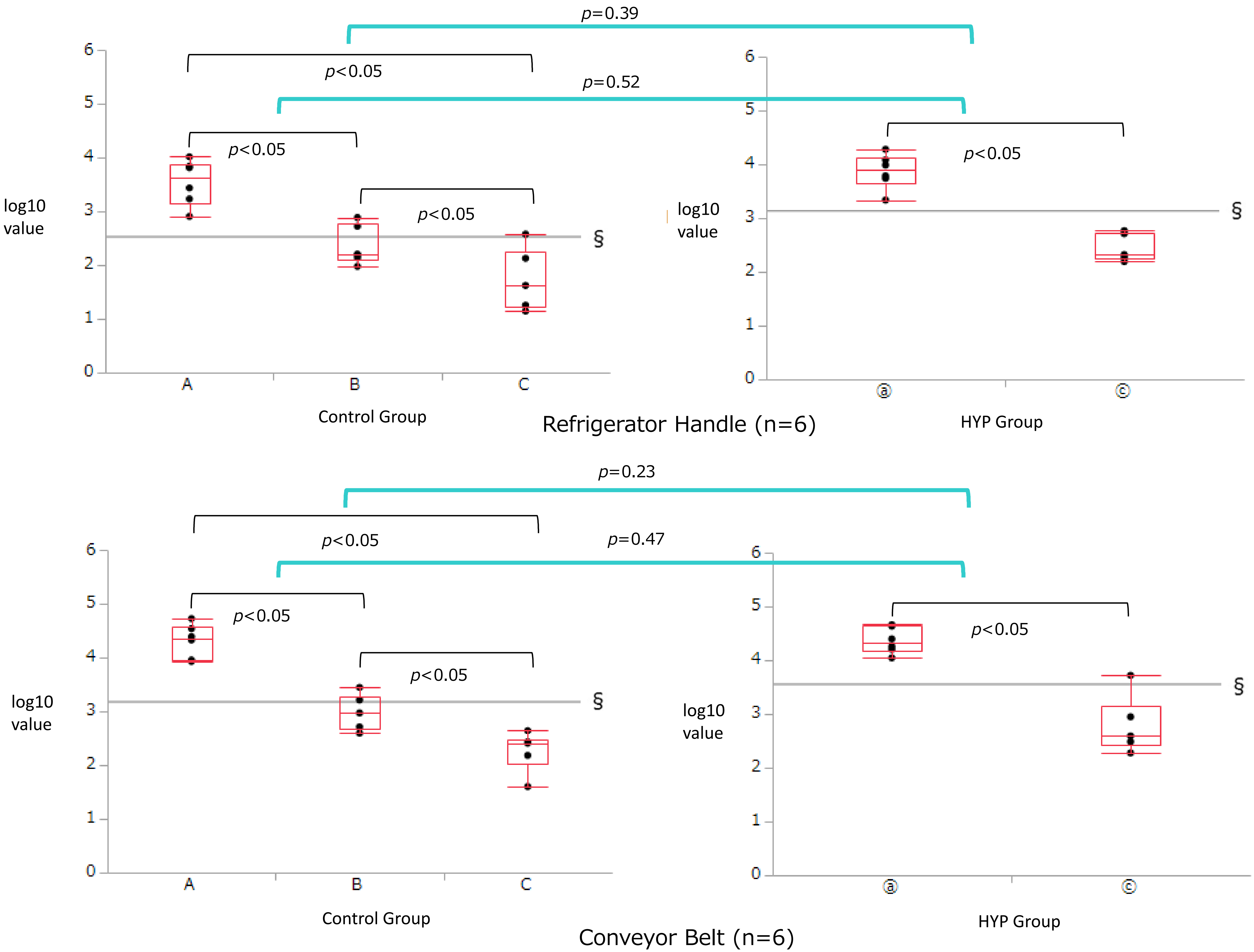

Fig. 2-2: Inter-group comparison of the log10 values in the 6 test locations measured

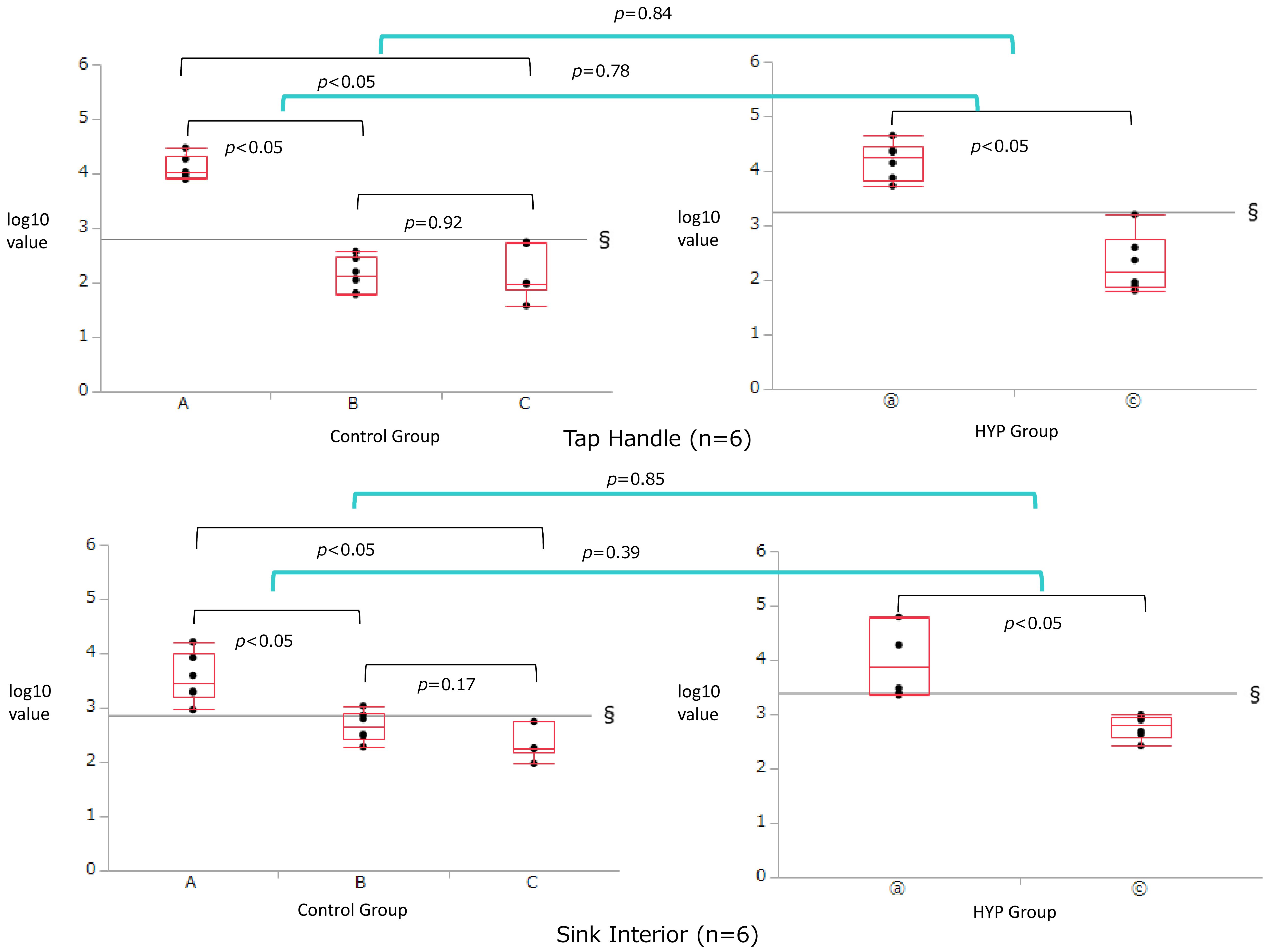

Fig. 2-3: Inter-group comparison of the log10 values in the 6 test locations measured
