# Supplementary material for: Comparison of the disinfecting effect of sodium hypochlorite aqueous solution and surfactant on hospital kitchen hygiene using adenosine triphosphate swab testing
Source: PLoS One. 2021 Apr 13;16(4):e0249796. doi: 10.1371/journal.pone.0249796 (PMC8043410; doi:10.1371/journal.pone.0249796)
Supplement: Attachment — Submitted filename: Response_to_Reviewers.plos_one.docx [file pone.0249796.s003.docx]

February 17, 2021

Joerg Heber

Editor-in-Chief

*PLOS ONE*

Dear Hans-Uwe Dahms, Ph.D.

Academic Editor

*PLOS ONE*

Dear Editor:

I wish to re-submit the manuscript titled “Comparison of the disinfecting effect of sodium hypochlorite aqueous solution and surfactant on hospital kitchen hygiene using adenosine triphosphate swab testing.” The manuscript ID is PONE-D-20-31068.

I express my appreciation to the reviewers for their thoughtful suggestions and insights on my manuscript. The manuscript has benefited from these insightful suggestions. I look forward to working with you and the reviewers to move this manuscript closer to publication in *PLOS ONE*.

The manuscript has been rechecked and the necessary changes have been made in accordance with the reviewers’ suggestions. The responses to all comments have been prepared and are given below. The revisions in the manuscript are highlighted in yellow. I hope that my responses and revisions to the manuscript are satisfactory.

Thank you for your consideration. I look forward to hearing from you.

Sincerely,

Takashi Aoyama,

Division of Nutrition

Shizuoka Cancer Center

1007 Shimonagakubo Nagaizumi-cho, Sunto-gun, Shizuoka 411-8777, Japan

Tel.: +81-55-989-5222

Fax: 81-55-989-5634

E-mail: [t.aoyama@scchr.jp](mailto:t.aoyama@scchr.jp)

**Responses to the reviewers’ comments**

**Additional editor’s comment:**

**Comment:** The work was conducted carefully and provides sufficient data. The flow of thought is convincing and the English is suitable.

Unfortunately, INTRODUCTION and DISCUSSION (of own RESULTS and those of others are not detailed at this point! It is suggested that the authors add more reflections around the many MORE disinfection methods (incl. NON-CHEMICAL), comparison with available protocols WORLDWIDE, and report more DISCUSSION about these issues. For example ATP- approach will NOT detecet VIRUSES and it should be critically evaluated whether the ATP endpoint for a bioassay is appropriate to monitor MICROBES as well as pathogenic molecules like viruses....Would fungi be equally assayed with ATP? A minimum of 25-30 REFERENCES would be necessary to provide an in depth DISCUSSION.

**Response:** Thank you for your insightful suggestion. Per your suggestion, I have revised the Introduction and Discussion sections to include more background information and in-depth discussion, respectively, in the revised manuscript.

The following sentence has been added in the Introduction section on page 3, lines 66–67 of the revised manuscript:

“It has been reported that ATP swab testing should be conducted in conjunction with media methods.”

The following paragraphs have been added in the Discussion section of the revised manuscript:

Page 14, line 233–page 15, line 242 “Viruses are vulnerable to heating at 85°C, but it is difficult to use boiling water because large equipment and conveyor belts have electrical systems. For this reason, disinfection methods other than HYP have been reported. Alcohol preparations may or may not have a disinfecting effect on viruses, therefore alcohols (especially, 1-propanol) are as effective as low concentrations (about 100–200 ppm) of sodium hypochlorite, so sprays are used to disinfect the clean facility environment. Although it has been reported that alcohols are effective against ultraviolet rays, gamma rays, and microbubbles, they are not suitable for large equipment and facilities. Owing to the aforementioned reports, only HYP is recommended for norovirus in Japan.”

Page 15, lines 243–251 “The findings of the present study, which demonstrated the potential of HYP for general cleaning purposes, were considered to be significant. However, in this study, no other medium method was employed along with ATP swabbing. The ATP approach is unable to detect viruses and its findings should be critically evaluated to elucidate whether the ATP endpoint for a bioassay would be appropriate to monitor microbes as well as pathogenic molecules like viruses. There is also a room for consideration as to whether the fungi can also be detected by ATP.”

I have also included more references in the revised manuscript for in-depth discussion. The revised manuscript has 26 references.

**Reviewer comments**

**Reviewer #1**

**Comment:** After examining the manuscript, I am of the view that it is not suitable for publication in current form. Although the work appears to have been conducted carefully, unfortunately data, experiments and discussion are not enough, hence, not suitable for publication in this journal.

It is suggested that the authors should add more experimental data of various disinfection methods, comparison with available protocols, and report more discussion.

**Response:** Thank you for your critical comment. As described in response to the additional editor’s comment, I have revised the Introduction and Discussion sections to include more background information and in-depth discussion, respectively, in the revised manuscript.

**Reviewer #2**

**Comment:** After examining the manuscript, I am of the view that it is not suitable for publication in current form, particularly since an IN DEPTH DISCUSSION and LITERATURE update is missing. The work was conducted carefully and provides sufficient data

for its objectives. The flow of thought is convincing and the English is suitable.

Unfortunately, INTRODUCTION and DISCUSSION (of own RESULTS and those of others are not enough as yet! It is suggested that the authors add more reflections around the many MORE disinfection methods (incl. NON-CHEMICAL), comparison with available protocols WORLDWIDE, and report more DISCUSSION about these issues.

**Response:** Thank you for your feedback on my manuscript. As described in response to the additional editor’s comment, I have revised the Introduction and Discussion sections to include more background information and in-depth discussion, respectively, in the revised manuscript.
